# Supplementary material for: Antimicrobial Resistance Patterns and Risk Factors Associated with Salmonella spp. Isolates from Poultry Farms in the East Coast of Peninsular Malaysia: A Cross-Sectional Study
Source: Pathogens. 2021 Sep 9;10(9):1160. doi: 10.3390/pathogens10091160 (PMC8470665; doi:10.3390/pathogens10091160)
Supplement: Supplementary file 1 [file pathogens-10-01160-s001.zip › pathogens-1328074-supplementary.pdf]

**Table S1.** Characteristics of 31 farmers /farms in Kelantan, Terengganu, and Pahang states, Malaysia.

| Variables | Household farms |
|---|---|
| **Demographic characteristics of the farmer** | |
| Age of farm manager (years), median (IQR) | 45( 39-54) |
| Gender | |
| Female | 5(16.1%) |
| Male | 26 (83.9%) |
| Education | |
| Primary | 9(29.0%) |
| Secondary | 14(45.2%) |
| College | 8(25.8%) |
| | |
| **Characteristics of 31 farms** | |
| Use of antibiotics | |
| Yes | 31 (100%) |
| No | 0 |
| Vaccines | |
| Yes | 31(100%) |
| No | 0 |
| Recent disease history | |
| Yes | 22(71.0%) |
| No | 9(29.0%) |
| Animal contact | |
| Yes | 2 (6.5%) |
| No | 29(93.5%) |
| Production system | |
| Broiler | 16(51.6%) |
| Layer | 5(16.1%) |
| Mixed | 10 (32.3%) |
| Management system | |
| Intensive | 16(51.6%) |
| Semi-intensive | 12 (38.7%) |
| Mixed | 3(9.7%) |
| Farm size | |
| Small | 10(32.3%) |
| Medium | 13(41.9%) |
| Large | 8(25.8%) |
| Flock origin | |
| Local | 3(9.7%) |
| Imported | 9(29.0%) |
| Both | 19(61.3%) |
| States | |
| Kelantan | 13(41.9%) |
| Terengganu | 7(22.6%) |
| Pahang | 11(35.5%) |
| Districts | |
| Bachok | 2(6.5%) |
| | |

| | |
|---|---|
| Kota Bharu | 3(9.7%) |
| Machang | 1(3.2%) |
| Pasir Mas | 2(6.5%) |
| Pasir Puteh | 2(6.5%) |
| Jeli | 3(9.7%) |
| Kuantan | 6(19.4%) |
| Maran | 1(3.2%) |
| Pekan | 4(12.9%) |
| Kuala terengganu | 3(9.7%) |
| Marang | 4(12.9%) |
| Sewage system | |
| Excellent | 11(35.5%) |
| Good | 17(54.8%) |
| Poor | 3(9.7%) |
| Visitors | |
| Yes | 19(61.3%) |
| No | 12(38.7%) |
| PPE | |
| Yes | 19(61.3%) |
| No | 12(38.7%) |
| Washing facilities | |
| Yes | 25(80.6%) |
| No | 6(19.4%) |
| Use of disinfectant | |
| Yes | 21(67.7%) |
| No | 10(32.3%) |
| Source of antibiotic | |
| Drug supplier | 20(64.5%) |
| Feed store | 11(35.5%) |
| Veterinary services | |
| Yes | 31(100%) |
| No | 0 |
| Source of feed | |
| Endogenous | 12(38.7%) |
| Exogenous | 16(51.6%) |
| Water source | |
| Surface water | 6(19.4%) |
| Bond water | 13(41.9%) |
| Pump water | 12 (38.7%) |

**Table S2.** Summary of prevalence of resistance to at least one antimicrobial and their associated risk factors.

| Risk Factors | No antimicrobial resistance (n = 24) | Resistance to at least one antimicrobial (n = 153) |
|---|---|---|
| Age | | |
| Young | 12(50%) | 74(48.4%) |
| Adult | 12(50%) | 79(51.6%) |
| Origin of the poultry | | |
| Local | 3(12.5%) | 9(5.9%) |
| Imported | 5(20.8%) | 68(44.4%) |
| Both | 16(66.7%) | 76(49.7%) |
| Management system | | |
| Intensive | 7(29.2%) | 88(57.5%) |
| Semi-intensive | 14(58.3) | 56(36.6%) |
| Mixed | 3(12.5%) | 9(5.9%) |
| Production System | | |
| Broiler | 13(54.2%) | 96(62.7%) |
| Layer | 1(4.2%) | 24(15.7%) |
| Mixed | 10(41.7%) | 33(21.6%) |
| Farm size | | |
| Small | 9(37.5%) | 41(26.8%) |
| Medium | 14(58.3%) | 83(54.2%) |
| Large | 1(4.2%) | 29(19%) |
| Source of sample | | |
| Cloacal swab | 20(83.3%) | 100(65.4%) |
| Faecal sample | 0 | 50(32.7%) |
| sewage | 2(8.3%) | 3(2%) |
| Tap water | 2(8.3%) | 0 |
| Water source | | |
| Surface water | 6(25%) | 32(20.9%) |
| Bond water | 7(29.2%) | 65(42.5%) |
| Pump water | 11(45.8%) | 56(36.6%) |
| Sewage system | | |
| Excellent | 4(16.7%) | 60(39.2%) |
| Good | 16(66.7%) | 76(49.7%) |
| Poor | 4(16.7%) | 17(11.1%) |
| Feed source | | |
| Endogenous | 8(33.3%) | 53(34.6%) |
| Exogenous | 15(62.5%) | 88(57.5%) |
| Other | 1(4.2%) | 12(7.8%) |
| Farms | | |
| Farm 1 | 0 | 16(10.5%) |

| | | |
|---|---|---|
| Farm 2 | 3(12.5%) | 9(5.9%) |
| Farm 3 | 3(12.5%) | 6(3.9%) |
| Farm 4 | 1(4.2%) | 12(7.8%) |
| Farm 5 | 5(20.8%) | 12(7.8%) |
| Farm 6 | 4(16.7%) | 8(5.2%) |
| Farm 7 | 0 | 7(4.6%) |
| Farm 8 | 0 | 12(7.8%) |
| Farm 9 | 1(4.2%) | 8(5.2%) |
| Farm 10 | 1(4.2%) | 17(11.1%) |
| Farm 11 | 3(12.5%) | 20(13%) |
| Farm 12 | 0 | 10(6.5%) |
| Farm 13 | 1(4.2%) | 7(4.6%) |
| Farm 14 | 2(8.3%) | 9(5.9%) |

**Table S3.** Number of samples per farm in east coast of peninsular Malaysia

| Farms | Cloacal swabs | Faecal | Sewage | Tap water |
|---|---|---|---|---|
| Farm 1 | 20 | 6 | 1 | 1 |
| Farm 2 | 18 | 6 | 1 | 1 |
| Farm 3 | 18 | 6 | 1 | 1 |
| Farm 4 | 18 | 6 | 1 | 1 |
| Farm 5 | 18 | 6 | 1 | 1 |
| Farm 6 | 18 | 6 | 1 | 1 |
| Farm 7 | 20 | 5 | 1 | 1 |
| Farm 8 | 18 | 7 | 1 | 1 |
| Farm 9 | 18 | 6 | 1 | 1 |
| Farm 10 | 18 | 6 | 1 | 1 |
| Farm 11 | 19 | 6 | 1 | 1 |
| Farm 12 | 18 | 6 | 1 | 1 |
| Farm 13 | 18 | 6 | 1 | 1 |
| Farm 14 | 20 | 6 | 1 | 1 |
| Total | 259 | 84 | 14 | 14 |

**Table S4.** The set of primers used for each gene.

| Genes | Primer sequence(5′to3′) | PCR condition | Product size | References |
|---|---|---|---|---|
| Tetracyclines *tetA* | F GTAATTCTGAGCACTGTCG<br>R CTGCCTGGACAACATTGCTT | 3 min at 94 °C; 30 cycles of 30 s at 94 °C, 30 s at 62 °C and 1 min at 72 °C; 7 min at 72 °C | 957 | [48] |
| Tetracyclines *tetB* | F CTCAGTATTCCAAGCCTTTG<br>R ACTCCCCTGAGCTTGAGGGG | 3 min at 94 °C; 30 cycles of 30 s at 94 °C, 30 s at 62 °C and 1 min at 72°C; 7 min at 72 °C | 414 | [49] |
| Sulfonamide *sul1* | F TCACCGAGGACTCCTTCTTC<br>R CAGTCCGCCTCAGCAATATC | 3 min at 94 °C; 30 cycles of 30 s at 94 °C, 30 s at 53 °C and 1 min at 72 °C; 7 min at 72°C | 331 | [50] |
| Sulfonamide *Sul2* | F GCGCTCAAGGCAGATGGCAT<br>R GCGTTTGATACCGGCACCCT | 3 min at 94 °C; 30 cycles of 30 s at 94 °C, 30 s at 53 °C and 1 min at 72 °C; 7 min at 72 °C | 293 | [48] |
| Chloramphenicol *cat1* | F CTTGTCGCCTTGCGTATAAT<br>R AACGGCATGATGAACCTGA | 10 min at 95 °C; 30 cycles of 30 s at 95 °C, 1 min at 55 °C and 1 min at 72 °C; 7min 72 °C | 508 | [50] |
| Chloramphenicol *Cat2* | F AACGGCATGATGAACCTGAA<br>R ATCCCAATGGCTCGTAAAG | 10 min at 95 °C; 30 cycles of 30 s at 95 °C, 1 min at 55 °C and 1 min at 72 °C; 7min 72 °C | 547 | [50] |
| Chloramphenicol *floR* | F CTGAGGGTGTCGTCATCTAC<br>R GCTCCGACAATGCTGACTAT | 10 min at 95 °C; 30 cycles of 30 s at 95 °C, 1 min at 55 °C and 1 min at 72 °C; 7min 72 °C | 673 | [50] |
| *blaTEM* | F ATGAGTATTCAACATTTCCG<br>R ACCAATGCTTAATCAGTGAG | 3 min at 94 °C; 25 cycles of 1 min at 94 °C, 1 min. at 50 °C and 1 min at 72 °C; 10 min at 72 °C | 857 | [49] |

**Table S5.** The biochemical characteristics and PCR confirmation of *Salmonella* spp.

| The biochemical characteristics of *Salmonella* spp | | |
|---|---|---|
| **Characteristics** | ***Salmonella*** | |
| Simmons citrate | +ve | |
| Urease | -ve | |
| Indole | -ve | |
| Methyl red | +ve | |
| Triple Sugar Iron (TSI) | Alkaline/Acid, gas | |
| $H_2S$ | +ve | |
| Lysine Iron Agar | +ve | |
| PCR confirmation of *Salmonella* spp. | | |
| **Primer sequence(5′to3′)** | **PCR condition** | **Product size** |
| F GTG AAA TTA TCG CCA CGT TCG GGC AA<br>R TCA TCG CAC CGT CAA AGG AAC C | 1 min at 94 °C; 35 cycles of 1 min at 94 °C, 30 s at 55 °C and 45 s min at 72 °C; 7min at 72 °C | 284 bp |
